# A semi-automated quantitative comparison of metal artifact reduction in photon-counting computed tomography by energy-selective thresholding

**DOI:** 10.1038/s41598-020-77904-3

**Published:** 2020-12-03

**Authors:** T. D. Do, S. Sawall, S. Heinze, T. Reiner, C. H. Ziener, W. Stiller, H. P. Schlemmer, M. Kachelrieß, H. U. Kauczor, S. Skornitzke

**Affiliations:** 1 Clinic for Diagnostic and Interventional Radiology (DIR), Heidelberg University Hospital, Im Neuenheimer Feld 420, 69120 Heidelberg, Germany; 2 Division of X-Ray Imaging and CT, German Cancer Research Center (DKFZ), Heidelberg, Germany; 3 Institute of Forensic and Traffic Medicine, Heidelberg University Hospital, Heidelberg, Germany; 4 Department of Orthopedics and Trauma Surgery, Heidelberg University Hospital, Heidelberg, Germany; 5 Department of Radiology, German Cancer Research Center (DKFZ), Heidelberg, Germany

**Keywords:** X-ray tomography, Skeleton

## Abstract

An evaluation of energy thresholding and acquisition mode for metal artifact reduction in Photon-counting detector CT (PCD-CT) compared to conventional energy-integrating detector CT (EID-CT) was performed. Images of a hip prosthesis phantom placed in a water bath were acquired on a scanner with PCD-CT and EID-CT (tube potentials: 100, 120 and 140 kV_p_) and energy thresholds (above 55–75 keV) in Macro and Chess mode. Only high-energy threshold images (HTI) were used. Metal artifacts were quantified by a semi-automated segmentation algorithm, calculating artifact volumes, means and standard deviations of CT numbers. Images of a human cadaver with hip prosthesis were acquired on the PCD-CT in Macro mode as proof-of-concept. Images at 140 kV_p_ showed less metal artifacts than 120 kV_p_ or 100 kV_p_. HTI (70, 75 keV) had fewer artifacts than low energy thresholds (55, 60, 65 keV). Fewer artifacts were observed in the Macro-HTI (8.9–13.3%) for cortical bone compared to Chess-HTI (9.4–19.1%) and EID-CT (10.7–19.0%) whereas in bone marrow Chess-HTI (19.9–45.1%) showed less artifacts compared to Macro-HTI (21.9–38.3%) and EID-CT (36.4–54.9%). Noise for PCD-CT (56–81 HU) was higher than EID-CT (33–36 HU) irrespective of tube potential. High-energy thresholding could be used for metal artifact reduction in PCD-CT, but further investigation of acquisition modes depending on target structure is required.

## Introduction

CT detector technology substantially influences image quality and radiation dose. Photon-counting detector CT (PCD-CT) uses the direct conversion of photons into electronic signals (“electron hole pairs”) instead of the indirect detection by conversion to light as in scintillators^1,2^. In contrast to conventional CT, the PCD-CT detector counts every photon that overcomes a specified photon energy threshold. Thereby, the energy information of each photon can be preserved. However, for the photon counting detector evaluated in this study, photons are sorted into discrete bins based on energy thresholds that have to be selected before the CT acquisition and cannot be retrospectively modified once the acquisition is completed. Depending on the vendor, two to eight energy thresholds can be employed at the same time. Therefore, a large number of combinations of specified photon energy thresholds is possible. Additionally, acquisition parameters such as tube potential and acquisition mode also influence image quality, for example the extent of metal artifacts. This large number of potential parameter combinations poses the problem of selecting optimal parameters to achieve the best available image quality.

One advantage of PCD-CT detector technology is the potential to reduce beam-hardening artifacts in comparison to energy-integrating detectors (EID) when using high-energy bins, as has been shown in a small animal scanner^3^. Metal artifacts can diminish image quality, reducing diagnostic accuracy and disguise important findings, and several approaches exist for the reduction of metal artifacts^4–7^. Recent studies recommend the use of high-energy thresholds and, if available, a tin filter in PCD-CT^1–3,8^. Given the potential advantages of PCD-CT technology for metal artifact reduction, the aim of the study was to evaluate the application of PCD-CT for the reduction of metal artifacts and to evaluate the effect of tube potential, energy thresholds and acquisition mode (Macro vs. Chess) on image quality and metal artifacts in in a whole-body research PCD-CT.

## Materials and methods

### CT scanner system

CT acquisitions were performed on a whole-body research PCD-CT (Somatom CounT; Siemens Healthineers, Forchheim, Germany) in which the PC detector is integrated in a dual-source, dual-detector configuration with a conventional EID. This results in a reduced field of view (FOV) of 27.5 cm for PCD-CT acquisitions. The detector pixels of the photon-counting detector consist of 4 × 4 subpixels, where each subpixel supports two energy thresholds: one low- and one high-energy threshold. The subpixels can then be configured for different acquisition modes. In this study, the Macro and Chess acquisition modes were evaluated. In Macro mode, the same thresholds are applied for all subpixels, effectively grouping all subpixels together. In Chess mode, the subpixels are assigned in an alternating manner (similar to a chess board) to two different threshold settings, thus allowing four energy thresholds (two high- and two low-energy), but at the cost of reduced dose efficiency, as each of the two threshold-settings can only use half of the x-rays incident on the detector^9^. Both acquisition modes had 16 cm z-coverage.

### CT acquisition and reconstruction

The titan femoral part of a total hip endoprosthesis (CLS Spotorno stem, Zimmer Biomet, Warsaw, IN, USA) was embedded into a foam cortical bone model (Sawbones, Vashon Island, Washington, USA), with a proximal maximal diameter of 5 × 4 cm und distal minimal diameter 3.2 × 3.6 cm. CT acquisitions of the phantom were performed in a water bath of 13 cm height and 22 cm width, in a craniocaudal orientation. Conventional image acquisition with EID was performed for tube potentials of 100, 120 and 140 kV_p_, as required for the so-called data completion scans. For PCD-CT acquisitions Macro and Chess mode were used as follows (tube potential/bin energy level): Macro 140/75, Macro 120/70, Macro 100/65, Chess 140/75, Chess 120/70, Chess 100/65, Chess 140/65, Chess 120/60, Chess 100/55, where Chess acquisitions at the same tube potential were performed simultaneously (Table 1). Tube current was adapted to achieve similar CTDI_vol_ of 20 mGy for all image acquisitions, which was chosen according to clinical routine protocols for the hip performed with automated exposure control. A collimation of 32 × 0.6 mm for EID and 32 × 0.5 mm for all PCD-CT was chosen to facilitate imaging at a similar dose efficiency according to vendor’s recommendation, based on the fact of decreasing dose efficiency with decreasing detector pixel size. Other acquisition and reconstruction parameters (pitch, reconstruction kernel, slice thickness and increment) were also kept constant between EID and PCD-CT scans (Table 1). Filtered back projection was chosen as the reconstruction method for both EID and PCD-CT acquisitions for comparability reason, as iterative reconstruction algorithms are not available for PCD-CT, yet. For PCD-CT only high-energy threshold images (HTI) were reconstructed, considering only photons above the selected threshold, e.g. for the acquisition Macro 140/75 only photons above 75 keV were considered. A slice thickness of 2 mm was selected to mirror clinical routine reconstructions, offering a compromise between decreased noise with larger slice thickness and the increased spatial resolution (in z-direction) with smaller slice thickness^10^.

**Table 1:** Acquisition protocol and reconstruction parameters for phantom and cadaver scans with energy-integrating detector CT (EID-CT), PCD-CT Macro mode and PCD-CT Chess mode.

Parameters	Phantom			Cadaver scans	
	EID-CT	PCD-CT Macro-HTI	PCD-CT Chess-HTI	EID-CT	PCD-CT Macro-HTI
Tube potential and energy threshold [kV_p_/keV]	140	140/75	140/75	140	140/75
			140/65		
	120	120/70	120/70		
			120/60		
	100	100/65	100/65		
			100/55		
CTDI_vol_	20 mGy	20 mGy	20 mGy	34.34 mGy	23.17 mGy
Pitch	0.6	0.6	0.6	0.6	0.6
Collimation	32 × 0.6 mm	32 × 0.5 mm	32 × 0.5 mm	32 × 0.6 mm	32 × 0.5 mm
Reconstruction kernel	B70f.	B70f.	B70f.	B70f.	B70f.
Slice thickness/increment	2/1 mm	2/1 mm	2/1 mm	2/1 mm	2/1 mm

Image acquisitions of the water bath without the hip phantom were also performed with identical acquisition and reconstruction parameters as reference with EID and PCD-CT.

### Cadaver imaging

Additionally, to demonstrate the clinical applicability of the obtained results, acquisitions of a human cadaver with a hip prosthesis performed at the PCD-CT were retrospectively evaluated in cooperation with the Institute of Forensic and Traffic Medicine. Image acquisitions of the human cadaver were carried out in accordance with all applicable regulations and guidelines, and with approval from the local ethics committee of the Heidelberg University Hospital. The need to obtain informed consent from the next of kin was waived by the ethics committee.

Image acquisition had been performed at 140 kV_p_ with EID-CT and PCD-CT Macro mode with an energy threshold of 75 keV, i.e. Macro 140/75. CTDI_vol_ for EID-CT was 34.34 mGy and 23.17 mGy for PCD-CT. Otherwise, acquisition and reconstruction parameters were identical to phantom acquisitions. HTI reconstructions of the Macro mode acquisition were used for comparison with EID images.

### Quantitative image analysis

Segmentation was performed with the freely available Medical Imaging Interaction Toolkit (version 2018.04.2, available from http://mitk.org/wiki/MITK)^11^. This software allows a three-dimensional segmentation of metal artifacts based on thresholds calculated from the image data, as previously published^12^. Segmentation was performed as follows, with a more detailed description available in Do et al.^12^:

1. Manual segmentation of the cortical bone, bone marrow and water bath in the artifact free distal femur (7.2 cm length in z-axis) was performed in the conventional images to be used as reference volumes to determine reference values for investigated materials.
2. Lower and upper thresholds were calculated for the artifact segmentation based on the mean CT number ± 3 standard deviations of the reference segmentation for each investigated material (cortical bone, bone marrow and water). All voxels with CT numbers above or below the thresholds will be considered artifacts (see also explanation below).
3. The volume of interest (VOI), i.e. where artifacts are measured, was defined as the femoral shaft containing the prosthesis with a z-axis length of 9 cm. Manual segmentation of the volume of interest in cortical bone, bone marrow and water bath was performed in the femoral part with artifacts in the first CT series (HTI of Macro 140/75).
4. Segmentation of artifacts in VOI was automatically calculated as the intersection of the VOI and all voxels with CT-numbers beyond the calculated thresholds.
5. The manually segmented VOI with artifacts in the femoral shaft from the first CT series was copied to all other CT series, as all CT series were acquired in the exact same position. Thus, subsequent segmentations of artifacts in the volumes of interest based on calculated thresholds (i.e. repetition of step 4 for all series) were able to maintain the comparability between series. The volume of the segmented artifacts was calculated.
6. The final percentage of artifacts for each series was calculated by dividing the calculated volume of segmented artifacts by the size of the VOI.

**Figure 1:**
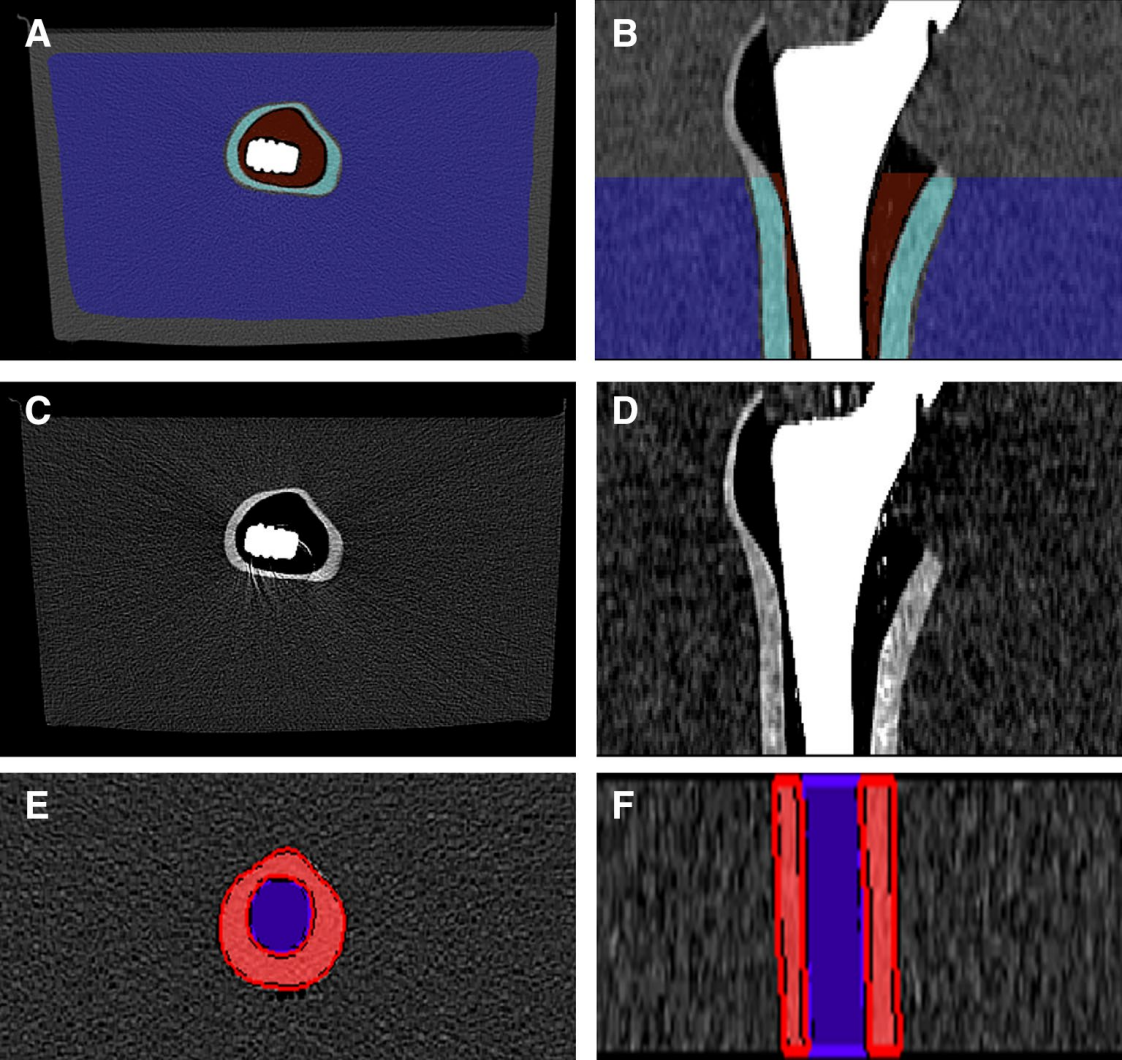
Femoral phantom with hip prosthesis placed in a water bath. Segmentation of volumes of interest of the bone marrow (brown), cortical bone (turquoise) and water bath (blue) in the axial plane (**a**) and coronal plane (**b**). For comparison, images without segmentation in the axial plane (**c**) and coronal plane (**d**). Segmentation of reference volumes in the distal femur without prosthesis in the cortical bone (red) and bone marrow (purple) in the axial plane (**e**) and coronal plane (**f**). Images were created with the freely available Medical Imaging Interaction Toolkit (version 2018.04.2, available from http://mitk.org/wiki/MITK)^11^.

**Figure 2:**
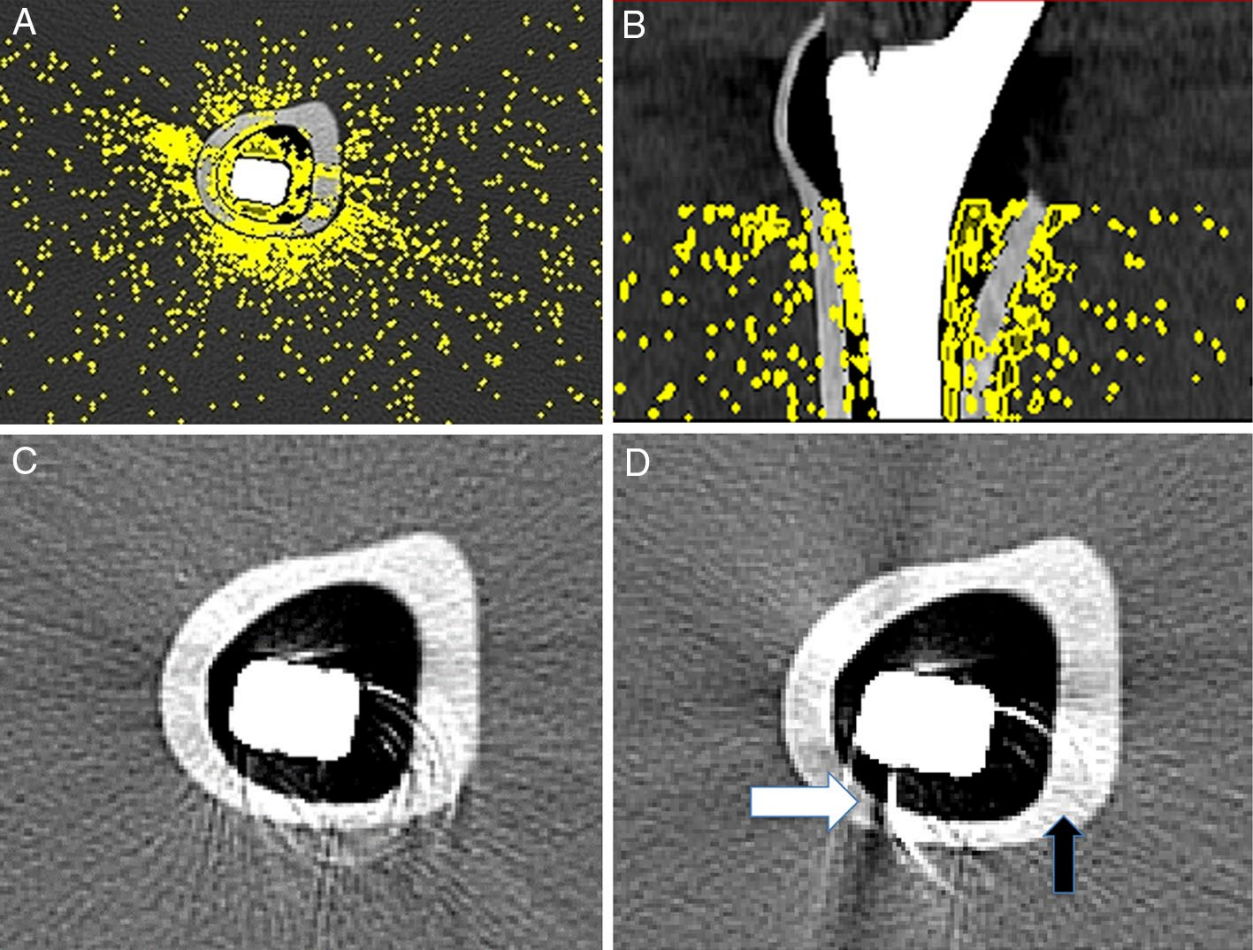
Femoral phantom with prosthesis embedded in a water bath and depiction of artifact volumes in the cortical bone, bone marrow and water color-coded in the axial plane (**a**) and coronal plane (**b**) on EID-CT at 120 kV_p_. Image examples of HTIs in Chess mode (**c**) and in Macro mode (**d**) highlight the increased artifacts in bone marrow (white arrow) and reduced artifacts in cortical bone (black arrow) for Macro-mode HTIs compared to Chess-mode HTIs. Images were created with the freely available Medical Imaging Interaction Toolkit (version 2018.04.2, available from http://mitk.org/wiki/MITK)^11^.

Artifact free reference volumes were chosen with at least 10 cm in distance to the slices where artifacts were clearly visible. The segmented reference volume had a size of 7.2 cm along the z-axis. A margin of 2 mm to the neighboring water and bone marrow was kept to avoid partial volume effects for the segmentation of the cortical bone. The same principle was applied for the segmentation of bone marrow with a 2 mm margin to the cortical bone and for the water bath with a 3 cm margin to the surface and to the container (Fig. 1). Conventional images, from a standard CT acquisition with the EID, were used to determine the thresholds for 100 kV_p_, 120 kV_p_ and 140 kV_p_ separately as the tube potential influences the measured CT numbers. The lower and upper thresholds were defined as mean CT numbers ± three standard deviations as determined in the artifact-free reference volumes^12^, which, assuming a normal distribution of CT numbers and no artifacts, should encompass 99.7% of normal tissue. Thresholds were separately determined for cortical bone/bone marrow/water bath CT based on their reference volumes and for each tube potential. CT numbers outside of the interval of mean ± three standard deviations were classified as metal artifacts and segmented automatically. The segmented volume of interest had a size of 9 cm along the z-axis. Segmentations of CT numbers outside of the thresholds were combined with segmented volumes of interest (i.e. an intersection was calculated) to calculate the amount of metal artifacts in the volume of interest (Fig. 2). To account for differences in the sizes of the volumes of interest between cortical bone, bone marrow and water bath, relative artifact percentages were calculated by dividing the volume of the artifacts by the size of the volume of interest. As previously shown, a correction factor has to be taken into account as approximately 0.3% of voxels will be incorrectly identified as artifacts^12^. The correction factor is necessary as the thresholds are determined for one reference acquisition (i.e. the EID acquisition) and the actual amount of incorrectly classified voxels might differ between acquisitions, e.g. because of noise or differences in reconstruction algorithms. The correction factor is calculated by applying the calculated thresholds to the artifact-free reference segmentation and measuring the amount of voxels classified as artifacts.

For the evaluation of the cadaver images, a segmentation of the cortical bone, bone marrow and muscle compartment of the left thigh was evaluated as a proof-of-concept. Regions of interest of PCD-CT images were transferred to EID-CT due to the smaller FOV of PCD-CT.

CT numbers and standard deviations of the segmented volumes were calculated from the complete 3D segmentation. Noise in the images was defined and measured as the standard deviation of the CT numbers measured in the segmentation of the water bath without phantom to ensure that no peripheral artifacts might distort results of the noise evaluation. For the calculation of the contrast-to-noise ratio (CNR) mean CT numbers of cortical bone and bone marrow were compared to those measured in the water bath (with phantom present) relative to the standard deviation of CT numbers in the water bath.

### Statistical analysis

Data were tabulated with Excel (Version 16.22, Microsoft Corporation, Redmond USA) and descriptive statistics (mean and standard deviation) were calculated.

## Results

Reference segmentations, volume of interest segmentations and segmentation of metal artifacts could successfully be performed as described for all acquisitions.

**Figure 3:**
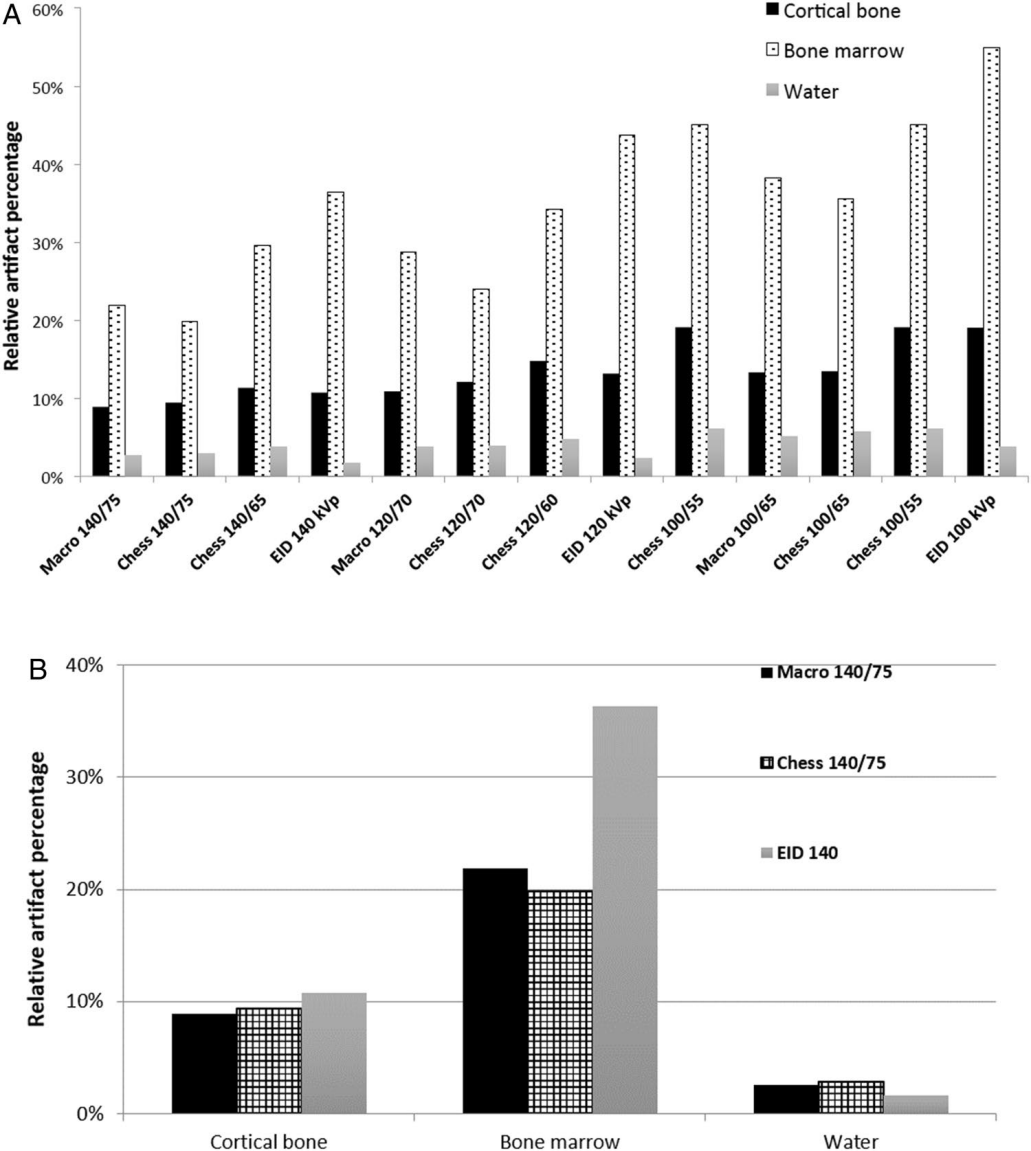
(**a**) Artifact percentage depending on tube potential and energy threshold. (**b**) Metal artifacts depending on location in high-energy threshold images with 140 kV_p_/75 keV compared to EID-CT.

Over all acquisitions a negative trend of artifact percentage based on the distance of the evaluated volume of interest to the prosthesis could be observed (Fig. 3a,b). Bone marrow (EID-CT: 36.4–54.9%; PCD-CT: 11.4–45.1%) had the highest percentages of metal artifacts, followed by cortical bone (EID-CT: 10.7–19%; PCD-CT: 6.7–19.1%) and water bath (EID-CT: 1.7–3.7%; PCD-CT: 2.0–6.1%). As expected, fewer artifacts were observed at increased tube potential, irrespective of acquisition mode.

Although the Macro-HTI artifact percentage for cortical bone (8.9–13.3% artifacts) was lower than for Chess-HTI (9.4–13.5% artifacts) at same tube potential and when using the same energy thresholds, Macro-HTI were not necessarily superior for all tissue compartments: for bone marrow, Chess-HTI (140/75: 19.9%; 120/70: 24.0%; 100/65: 35.6%) showed less artifacts than the equivalent setting in the Macro-HTI (140/75: 21.9%; 120/70: 28.8%; 100/65: 38.3%) (Fig. 3a). Otherwise, Macro-HTI showed reduced artifacts compared to Chess-HTI. The Macro-HTI were superior with regard to metal artifacts to EID-CT acquisitions for cortical bone and bone marrow (Fig. 3b). Less artifacts were observed for all Chess-HTI than in corresponding conventional images of EID-CT for all acquisition settings in bone marrow. However, Chess-HTI acquired with lower energy threshold showed more metal artifacts than corresponding EID-CT images for cortical bone and water bath. In the image periphery, as represented by the water bath, less artifacts were observed for the EID-CT in comparison to all evaluated PCD-CT HTI.

Regarding measured CT numbers, setting high keV thresholds leads to lower CT numbers in PCD-CT images as low-energetic photons are no longer considered for image reconstruction (Table 2). When using the same thresholds, CT numbers for Chess-HTI differed from those observed for Macro-HTI. The mean of CT numbers of the water bath with and without phantom was consistently around 0 HU for all acquisitions with a maximum difference of 1.7 HU for the water bath with phantom and 1.6 HU for water bath only (without phantom).

**Table 2:** Mean and SD of CT numbers of high-energy threshold images in cortical bone, bone marrow, water bath and water bath only.

Scan mode, kV_p_ and keV	Cortical bone		Bone marrow		Water bath with phantom		Water bath only	
	Mean [HU]	SD [HU]	Mean [HU]	SD [HU]	Mean [HU]	SD [HU]	Mean [HU]	SD [HU]
Macro-HTI 140/75	595.96	125.94	− 647.91	112.03	− 0.03	56.87	0.18	55.23
Chess-HTI 140/75	614.68	123.64	− 661.37	119.87	0.38	80.12	0.02	77.25
Chess-HTI 140/65	607.59	141.91	− 632.69	117.38	1.70	65.26	1.34	63.21
EID-CT 140	679.70	80.93	− 674.12	81.22	− 0.34	34.62	− 0.27	32.95
Macro-HTI 120/70	615.25	139.17	− 641.32	111.60	1.08	57.10	1.60	55.62
Chess-HTI 120/70	640.22	124.91	− 665.98	121.25	0.73	79.89	0.26	76.92
Chess-HTI 120/60	652.25	128.28	− 650.65	115.25	0.36	62.67	− 0.09	60.28
EID-CT 120	711.29	84.67	− 676.49	81.12	− 0.36	34.60	− 0.12	32.80
Macro-HTI 100/65	652.69	141.56	− 645.39	104.58	0.54	57.81	0.29	55.53
Chess-HTI 100/65	671.94	142.05	− 665.54	120.07	0.09	80.84	0.17	77.37
Chess-HTI 100/55	709.94	121.90	− 667.90	104.16	− 0.78	61.96	− 0.66	59.24
EID-CT 100	761.15	86.63	− 679.67	80.87	− 0.29	35.57	0.35	33.64

**Figure 4:**
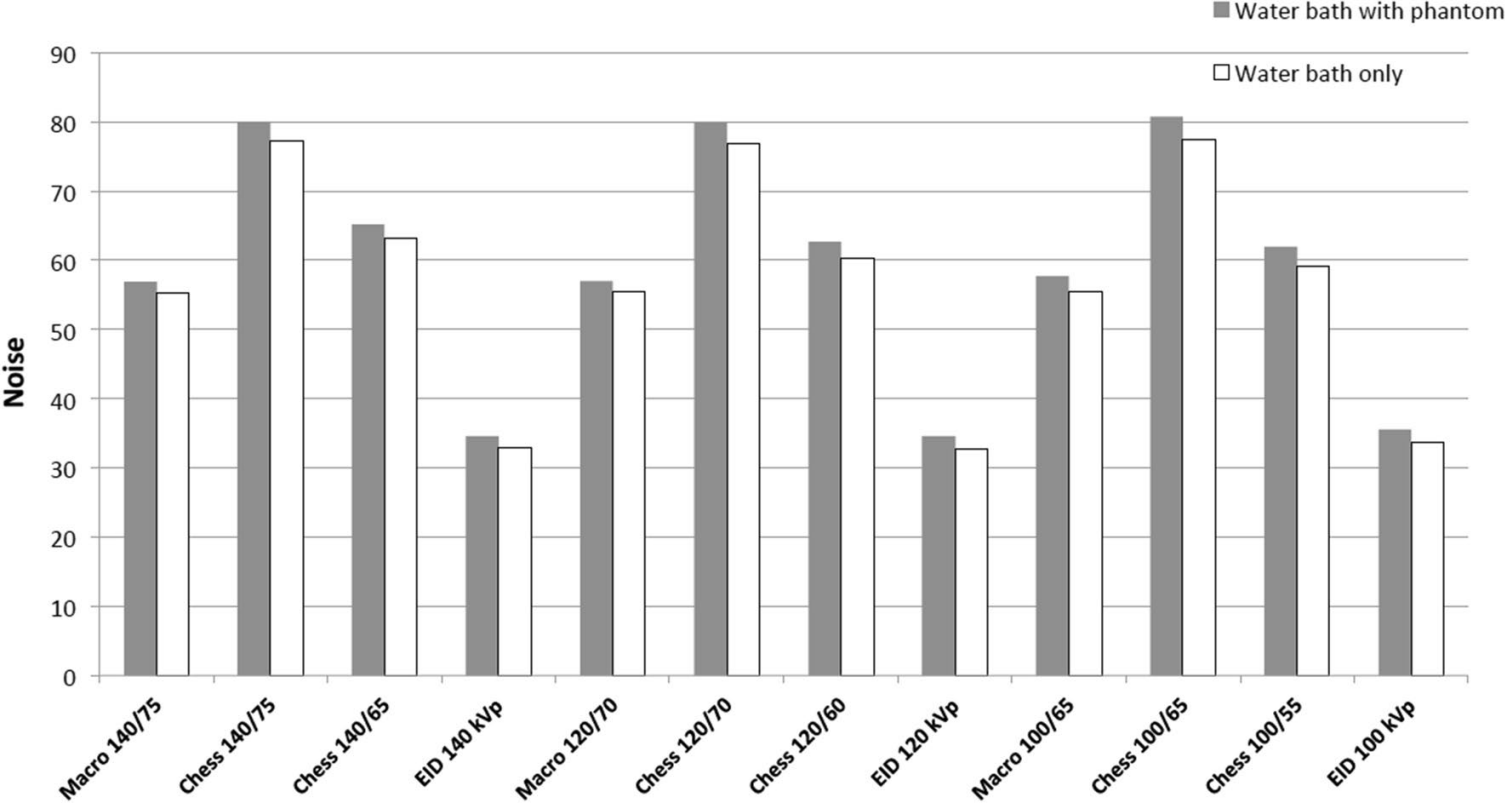
Noise depending on tube potential and energy threshold.

Increased noise was observed on all images of the water bath with the phantom in comparison to water bath only (Fig. 4). The differences in noise were increased in images acquired with lower tube potential. Conventional images (33–36 HU) showed less noise than all HTI in Macro and Chess mode (56-81HU). In general, noise was less in Macro-HTI than in Chess-HTI irrespective of tube potential and energy thresholds. CNRs were highest for EID-CT images at all energy levels. CNR was higher for HTIs in Macro mode than in Chess mode when comparing images with the same acquisition settings, with the exception of the acquisition at 120 kV_p_/70 keV (Table 3).

**Table 3:** Contrast-to-noise ratio (CNR) when comparing CT numbers of cortical bone and bone marrow to those measured in the water bath with phantom.

Scan mode, kV_p_ and keV	Cortical bone versus water	Bone marrow versus water
	CNR [HU]	CNR [HU]
Macro-HTI 140/75	6.10	7.29
Chess-HTI 140/75	5.90	6.49
Chess-HTI 140/65	5.49	6.68
EID-CT 140	10.93	10.79
Macro-HTI 120/70	5.77	7.25
Chess-HTI 120/70	6.10	6.49
Chess-HTI 120/60	6.46	7.02
EID-CT 120	11.00	10.84
Macro-HTI 100/65	6.03	7.64
Chess-HTI 100/65	5.81	6.50
Chess-HTI 100/55	7.35	7.78
EID-CT 100	11.50	10.88

**Figure 5:**
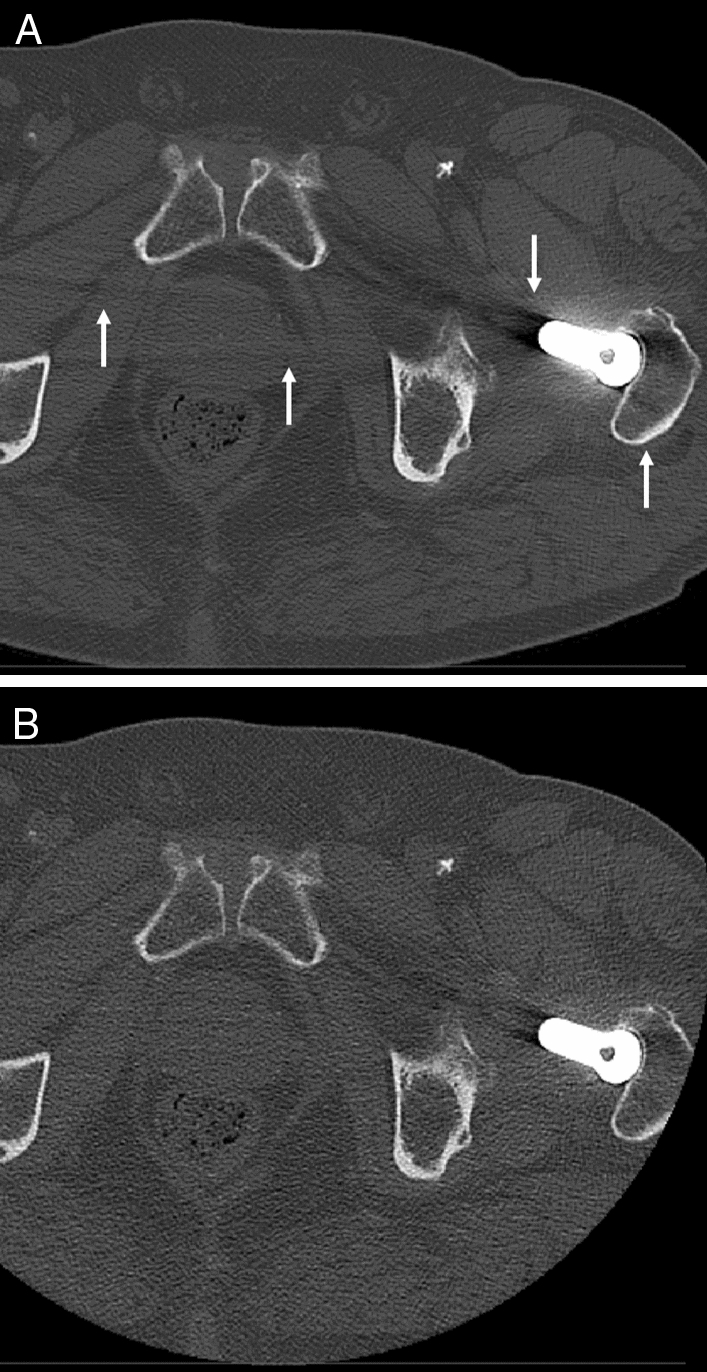
Axial CT of the pelvic region with prosthesis in the left hip. Artifacts in cadaver scans in EID-CT (**a**) and PCD-CT high-energy threshold Macro at 140kVp/75 keV (**b**). Note the increased metal artifacts in EID-CT in comparison to PCD-CT (white arrows: streak artifacts at the level of the femoral hip prosthesis). However, PCD-CT showed increased image noise leading to an increased number of “false metal artifacts”, subtracted by the correction factor. Images were created with the freely available Medical Imaging Interaction Toolkit (version 2018.04.2, available from http://mitk.org/wiki/MITK)^11^.

In the cadaver images, PCD-CT Macro-HTI showed fewer artifacts compared to EID-CT for cortical bone (18.3% vs. 27.6%) and bone marrow (35.3% vs. 60.6%) (Fig. 5). In the muscle compartment, Macro-HTI showed more artifacts than EID-CT (15.5% vs. 4.5%) if not corrected for noise (Fig. 5). After correction of misclassified artifact percentage the calculated artifact volume PCD-CT Macro-HTI (0.1%) is less than EID-CT (3.4%).

## Discussion

In PCD-CT, tube potential, energy thresholding and acquisition mode can influence the extent of metal artifacts and image noise. Metal artifacts can be reduced in the acquisition stage and it has already been suggested to use high-energy bins to diminish beam hardening artifacts in small animal PCD-CT^3^. Zhou et al. have suggested that high-energy thresholds are suitable to reduce metal artifacts in combination with a tin filter, which additionally removes low energy photons contributing to beam hardening artifacts^13,14^. Dual-energy acquisitions also make use of high-energy acquisitions to minimize metal artifacts, but might come along with higher radiation exposure^15^.

The results of this study show that high tube potential acquisitions with matched radiation dose and high keV thresholds can reduce metal artifacts compared to conventional EID-CT, but come at the price of increased image noise, as low energetic photons no longer contribute to image reconstruction.

Though Macro-HTI show good metal artifact reduction qualities compared to conventional images of EID-CT, Macro mode and Macro-HTI cannot be generalized to be the best acquisition mode or reconstruction algorithm. At the same energy thresholds, Chess-HTI surprisingly seem to perform better with less artifacts in the bone marrow bordering the prosthesis, but with increased image noise and reduced CNR. If this behavior of the acquisition modes can be verified in further studies, Macro-HTI could become the preferred method for imaging the cortical bone and periphery, whereas Chess-HTI could be more advantageous for bone marrow e.g. identification of prosthesis loosening if the image quality is sufficient. In this study only Chess-HTI and Macro-HTI were used. Ultra-high resolution mode and Sharp mode were not evaluated because of the prolonged acquisition times and might also be investigated in further studies.

Although the acquisitions were performed at the same tube voltage, CT numbers of Chess-HTI differed from those measured in Macro-HTI in cortical bone and bone marrow. This effect might be attributed to the high standard deviation of the measurement or to physical effects like the differences in x-ray tube position. However, the effect of these differences on the metal artifact quantification should be limited by the correction factor, which is calculated from the artifact-free reference segmentation. Nonetheless, the observed reduction in metal artifacts for Chess-HTI over Macro-HTI in bone marrow might be based on these differences in mean CT numbers and image noise, as there is no technical explanation as to why Chess-mode acquisitions should outperform Macro-mode acquisitions with regard to metal artifacts. This result illustrates that further research is necessary to fully understand the implications of photon counting acquisitions for clinical imaging. Future studies will have to investigate the observed change in mean CT numbers and validate the results on metal artifact reduction obtained in this study.

The differences in noise measured in the water bath with the phantom compared to the water bath only might be explained by the metal artifacts introduced by the prosthesis as well as by the absorption of the phantom, which might be even more pronounced in a clinical setting with higher patients’ body volumes. The advantage of reduced noise using low energy thresholds observed in other studies cannot be applied when imaging prosthesis at high energy thresholds and reconstructing high-energy threshold images^16^. Instead, increased image noise is observed when using high-energy thresholds for metal artifact reduction, because less photons are considered for image reconstruction. Furthermore, the increase in image noise for Chess-HTI compared to Macro-HTI can be explained by Chess mode using only half of the detector pixels compared to Macro mode which results in a predicted increase of image noise by the square root of 2, or approximately a factor of 1.4 for Chess mode over Macro mode, which becomes especially noticeable in the water bath scans. Similarly, CNR was diminished for HTIs in Chess mode compared to Macro mode with the exception of the acquisition at 120 kV_p_/70 keV. HTIs in general showed reduced CNR compared to EID images, which can be attributed to the higher noise HTIs and displays the trade-off between metal artifact reduction and image quality. In the future, the increase in noise for high-energy threshold images might be diminished by the use of iterative reconstruction algorithm routinely used with EID scanners^17,18^. Another possibility specific to PCD-CT is the reconstruction of two different images from one acquisition (and therefore without additional radiation exposure to the patient): one with a high energy threshold for metal artifact reduction and one with a low energy threshold for noise reduction^1^. These images could then be used in post-processing as the input for metal artifact reduction algorithms. In general, advantages of PCD-CT detector technology are expected to be more pronounced in a clinical setting, where the examined volumes in patients are larger than the phantom evaluated here.

The pelvic region has the highest diameter in the body, with the two hip joints causing potential photon starvation, leading to low detector signal intensity and increased noise. In the cadaver evaluated here, Macro-HTI showed reduced metal artifacts for cortical bone and bone marrow than EID-CT. In comparison to the phantom experiments, the increased amount of metal artifacts can be attributed to the effect of photon absorption and tissue inhomogeneity.

In this study, the evaluation was focused on a phantom, which has to be considered a limitation of this study. However, a similar investigation using human subjects would be unethical because of the repeated irradiation for comparison of different acquisition protocols. Moreover, the feasibility of the method was shown in a PCD-CT acquisition of a cadaver and can be investigated in further studies. The artifact segmentation was transferable to the cadaver acquisition and HTI reconstructions with selective evaluation of metal artifacts in the different compartments for the comparison of EID images and Macro-HTI. As the clinical transfer was performed retrospectively on a cadaver study the CTDI_vol_ was not matched because of the different scopes of the studies. Though the 140 kV_p_/75 keV Macro-HTIs acquired of the cadaver as a proof of principle with the PCD-CT showed fewer artifacts than the conventional EID-CT at 140 kV_p_, it would be also interesting to compare Macro and Chess mode and different acquisition settings for human bodies. Further clinical studies are needed to accurately assess the potential of the different settings for metal artifact reduction.

In contrast to other studies, lower photon energy thresholds were not examined in this study, as the focus was on metal artifact reduction. Moreover no subjective image quality was assessed in this study, as no actual pathology was available to base the assessment on. Therefore, further evaluation in a clinical setting seems necessary. Moreover, future studies will have to perform additional investigations on the observed differences between Macro and Chess mode regarding both measured CT numbers and metal artifacts.

## Conclusions

In summary, this study illustrates that metal artifacts can be reduced by using Photon-counting CT in combination with high-energy thresholds instead of energy-integrating detectors. Furthermore, differences between acquisition modes were observed for metal artifact reduction in different target structures: Macro-HTI showed better results for cortical bone while Chess-HTI seemed to produce fewer artifacts for bone marrow but at further increased image noise. Further investigation of the influence of the acquisition mode seems necessary.
